# More Light

**Published:** 1887-07-23

**Authors:** 


### "More Light."

"More light" and less gas is the last verse in the last chapter of the story of the lights. The Wenham Company claim, and not without reason, to be able to give us more light than we now get from three thousand feet of gas by the consumption of one thousand feet. Our gas bills are to be reduced from thirty pounds a year to ten. Most welcome news, and apparently as true as new. By the simple principle of heating beforehand the air which supplies the flame, all the gas is perfectly combusted, with the result above indicated. It might be supposed that the increase of light is accompanied by an increase of heat in the room: but that is not so, the heat of combustion being utilised in raising the temperature of the cold air before it passes to the flame. There is thus not only less gas consumed, but perfect combustion, with its consequent diminution of the products of combustion, and less heat given off into the room. A simple system of ventilation connected with the lamp carries off not only such waste heat as there may be, but also the combustion products as well, leaving the atmosphere of the room almost ideally pure. Anyone interested in the light problem, and still more any large or small user of artificial light, will certainly find it both interesting and profitable to pay one or two visits to the Wenham Light Company's works in Ogle Street, Fitzroy Square.

